# Kidney triplication with ectopic ureterocele: a case report

**DOI:** 10.1186/s12894-020-00625-2

**Published:** 2020-05-13

**Authors:** I. B. Osipov, D. A. Lebedev, M. V. Lifanova

**Affiliations:** Urology Department, State Pediatric Medical University, 2 Litovskaya Street, build.2, St. Petersburg, Russian Federation 194100

**Keywords:** Triplex kidney, Ureterocele, Vesicoureteral reflux, Incontinence

## Abstract

**Background:**

Kidney triplication is a rare urological abnormality. Association of triplex kidney and ureterocele is out of ordinary. Treatment of such patients usually implies heminephrureterectomy of the upper moiety. We report a case of a saved function of the upper moiety after minimal invasive surgical procedure.

**Case presentations:**

5-year old girl complained for continuous wetting. Examination revealed 3 - segmented left kidney with pelvi-ureteric dilation of the upper moiety, IV grade vesicoureteral reflux in the upper moiety, cervical ectopic ureteral orifice of the upper moiety and a commune ureteral orifice of the lower segments.

An endoscopic laser dissection of ureterocele was performed. Drainage of the upper moiety of triplex kidney was restored. Examination 18 months later showed no wetting and infection symptoms. Pelvi-ureteric dilation of the upper moiety and cavity of ureterocele decreased to minimal. Grade of vesicoureteral reflux decreased to I.

**Conclusion:**

Minimal invasive elimination of obstruction of the upper moiety of triplex kidney was successful and led to regress of vesicoureteral reflux, urinary incontinence and let to avoid heminephrectomy.

## Background

About 100 cases of renal triplication are described in scientific literature. A little over than 20 cases is associated with ureterocele. In 1870 Wrany first described triplex kidney with ureterocele that was revealed at 3-year-old girl autopsy [1, 2]. As usual ectopic ureterocele is associated with severe or critical damage of the upper moiety of triplex kidney.

Treatment of such patients usually implies heminephrureterectomy of the upper moiety [2, 3]. In case of extant function of the upper moiety there are some ways of minimal invasive treatment of obstruction. Mainly they are linked with the use of laser or electrocatheter for punction or dissection of ureterocele and upper segment decompression [4, 5]. We demonstrate successful outcome of endoscopic treatment of ectopic ureterocele in a child with kidney triplication.

## Case presentation

In august, 2017 5-year-old girl was admitted at pediatric urology department of Saint-Petersburg State Pediatric Medical University. The complaints were continuous wetting and recurrent urinary tract infection (UTI). Prenatal 32 weeks ultrasound revealed left pelvi-ureteric dilation. The child did not have episodes of anoxia, trauma, dehydration, hereditary pathology. By the age of 1 year the child had got over several episodes of UTIs.

Аt 1 year 5 months the child was examined at a local hospital. Ureterocele was revealed and an attempt to decompress it by endoscopic electric dissection was undertaken. At that time kidney triplication hadn’t been revealed. A diagnose sounded as complete left kidney duplication.

**Fig. 1:**
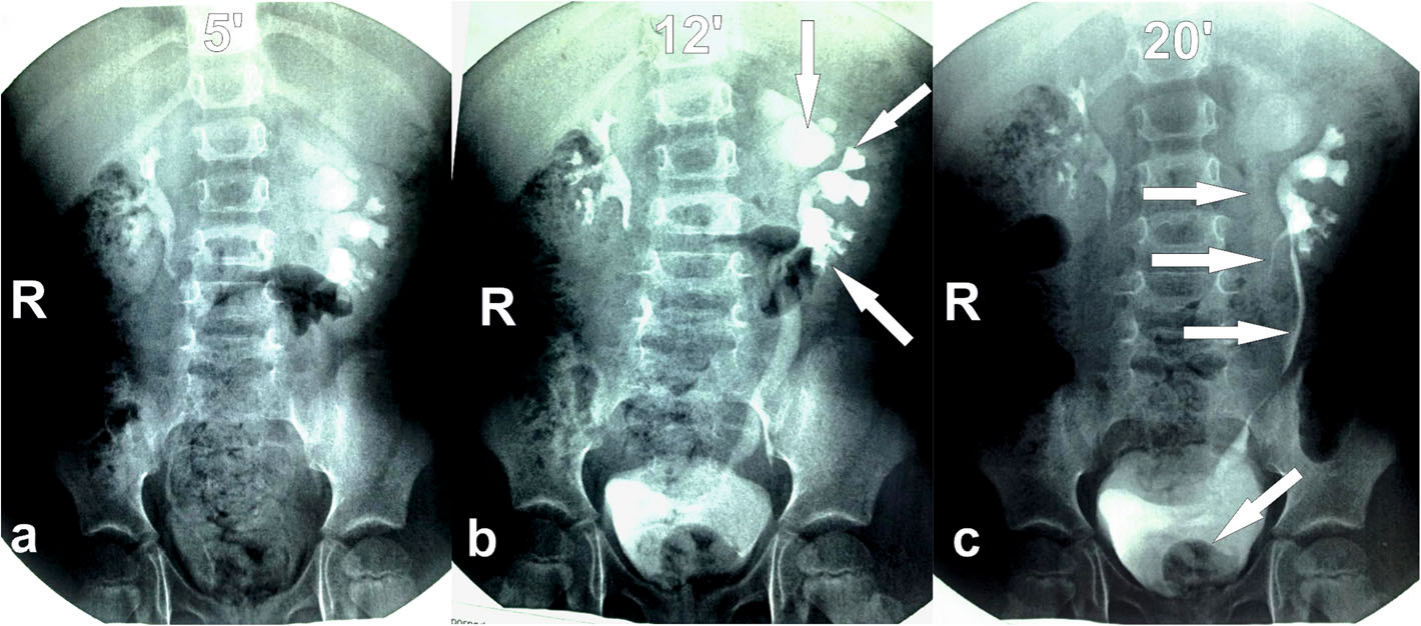
Intravenous pyelogram in 5 (**a**), 12 (**b**) and 20 (**c**) minutes after the injection of contrast media revealed left kidney triplication. Arrows indicate three segments of left kidney (IVPb) and three ureters (IVPc). The ureters of the middle and lower moieties merge along the way (IVPc)

Further examination and treatment were carried out at the base of Saint-Petersburg State Pediatric Medical University. Ultrasound revealed that left kidney consisted of 2 moieties with the upper moiety pelvis dilation to 30 × 15 mm. A large ureterocele was seen in the bladder. Intravenous pyelogram (IVP) demonstrated 3 contrasted moieties of the left kidney with megaureter of the upper one. Ureters of the middle and lower moieties fused along the way (Type 2 by Smith) [1]. There was rounded contrast defect in the bladder – ectopic ureterocele (Fig. 1).

**Fig. 2:**
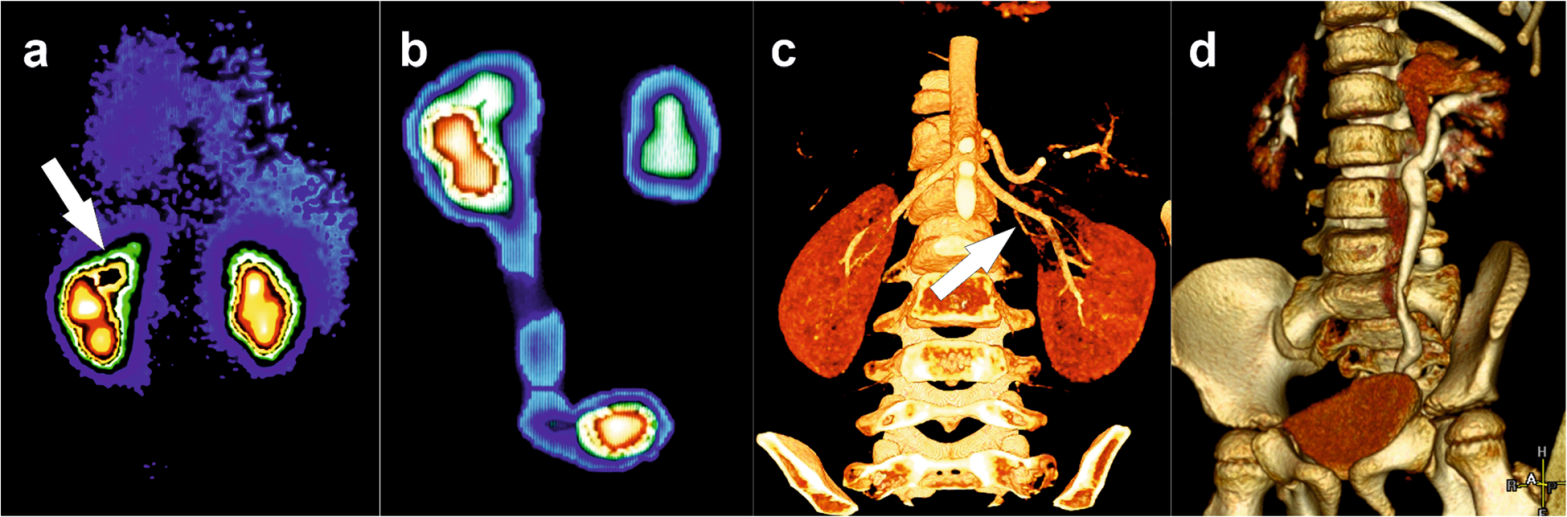
DMSA scans (**a** and **b**) show decrease of functioning parenchyma volume and urinary retention in the upper moiety. CT (**c** and **d**) show an additional vessel to the upper moiety (arrow), renal triplication and dilation of pelvicalyceal system and ureter of the upper moiety

DMSA scan confirmed extant function and urinary retention in the upper moiety. Functional capacity distributed as 51% to the right kidney and 49% to the left. Left upper moiety took upon itself 23% of left kidney function, middle and lower moieties – 77%. Computer tomography (CT) showed an additional blood supply of the upper moiety of triple kidney. The collecting system of the upper moiety and the ureter were significantly expanded (Fig. 2).

**Fig. 3:**
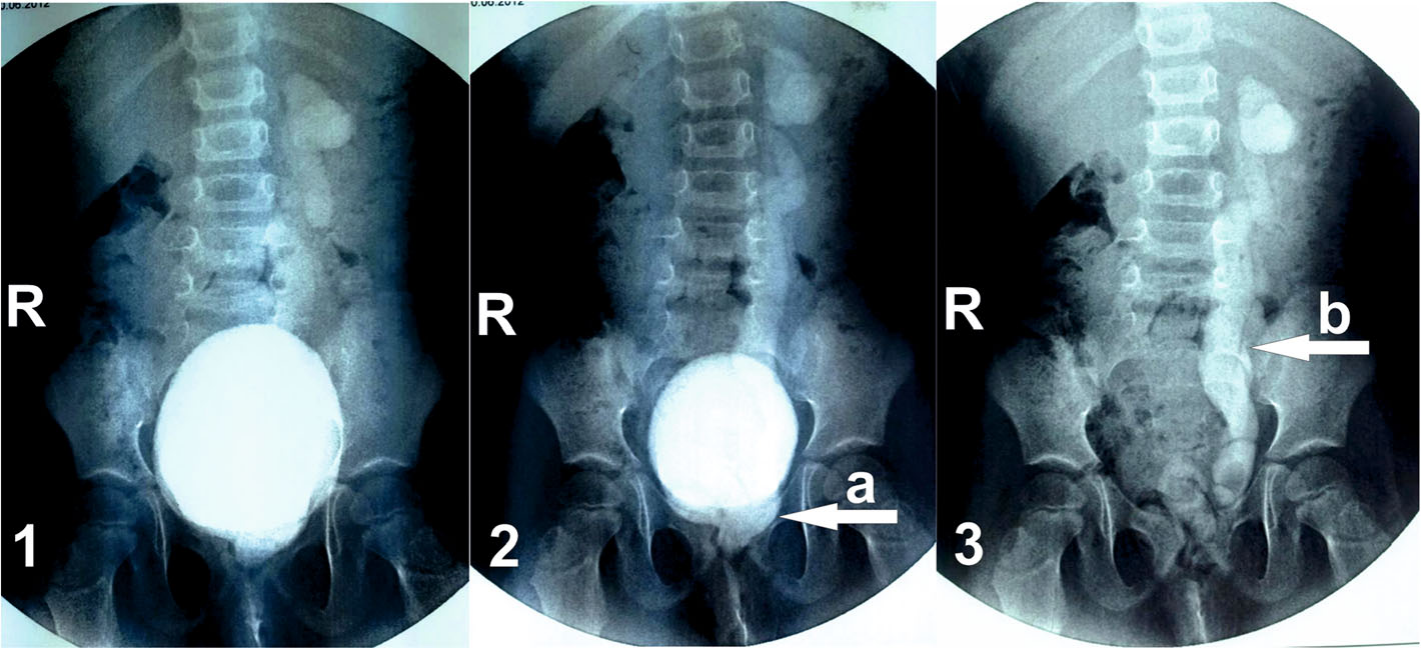
Voiding cystography: IV grade vesicoureteral reflux in the upper moiety. Contrast-filled ureterocele (**a**) and dilated ureter (**b**) are seen

Cystography revealed IV grade vesicoureteral reflux (VUR) in the upper moiety. Dilated pelvis, ureter and ureterocele were seen contrasted (Fig. 3).

In September, 2017 cystoscopy and laser dissection of ureterocele were performed. We used 10 Fr cystoscope, 4 Fr light guide, 10 W fiber tome mode Yag-Ni laser. Cystoscopy showed ectopic ureteral orifice of the upper moiety in the bladder neck, and a commune ureteral orifice of the lower segments. Ectopic orifice was wide and located in the sphincter zone. Ureterocele was large and located in the lower part of the bladder and it’s neck. We saw no traces of the previous surgical treatment and ureteral orifice hadn’t been deformed. We did not insert a catheter into the ureter since identification was not difficult and there was no risk of injury. A new upper moiety ureteral orifice was formed with the aid of laser at the front surface of ureterocele from the inside of it and then slightly widen from the inside of the bladder. Sufficient decompression of the upper moiety pelvi-ureteric system was achieved. There were no complications during surgery. Upper moiety was not catheterized. Bladder was catheterized with Foley catheter for a day. There were no complications during early postoperative period.

Complex investigation was performed in 7 months. UTI had no one episode by that time. Urinary incontinence considerably decreased. Ultrasound revealed much smaller dilation of the upper moiety pelvi-ureteric system. The pelvis size was 15 × 10 mm. Cavity of ureterocele was reduced to 5 mm in diameter. Cystography revealed II grade VUR in the upper moiety. Cystoscopy was performed to assess the local situation after surgery. A new formed upper ureteral orifice was able to discharge urine. There was no stenosis or inflammation.

**Fig. 4:**
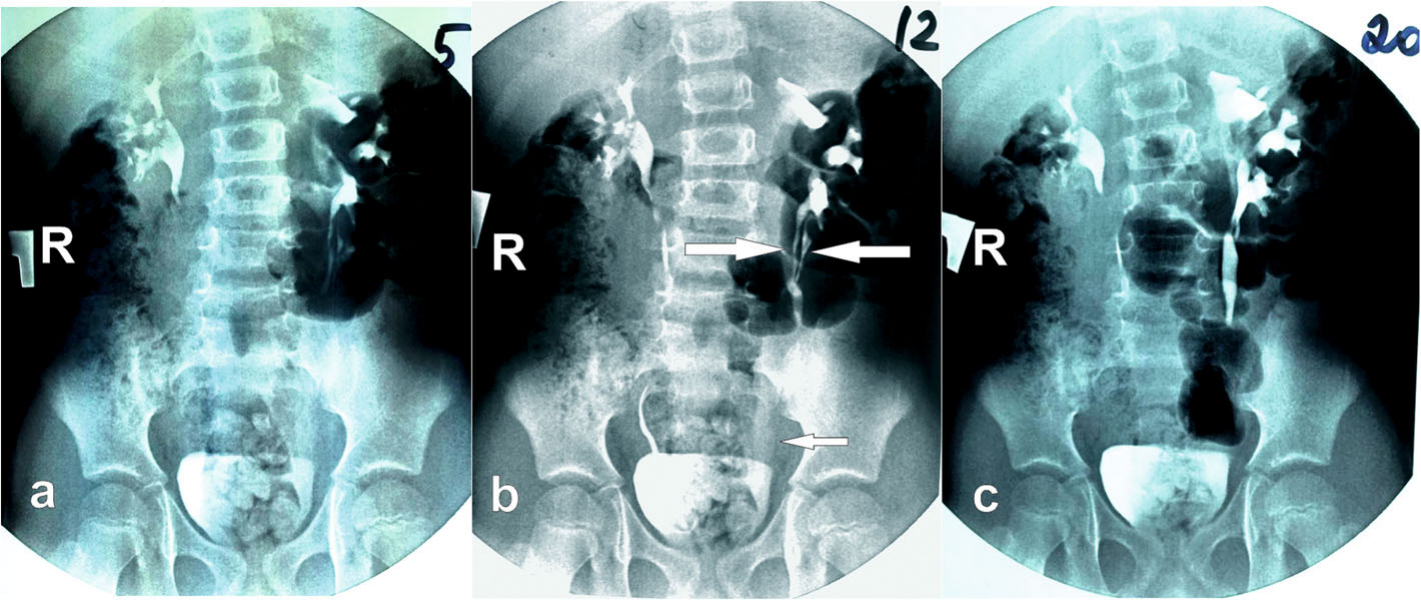
Intravenous pyelogram in 18 months after endoscopic surgery revealed a significant reduction of the ureter and pelvicalyceal system of the upper moiety and improved drainage of the middle and lower moieties

Investigation 1 year 6 months after surgery showed good results. There were no complaints for UTI symptoms. Urinalyses were good and culture showed no growth. Voiding was uncomplicated and there was no wetting. Uroflowmetry showed normal voiding without residual. Ultrasound demonstrated 10 mm dilation of the upper moiety pelvis. Parenchyma was rather thinned. There was 4 mm in diameter residual cavity of ureterocele in the bladder. Upper moiety ureter was 6 mm in the distal part. IVP confirmed positive dynamics in the upper moiety dilation (Fig. 4). Grade of VUR decreased to I. DMSA scan demonstrated decreased parenchymal mass of the upper moiety but improvement of absorption and transport function. We expect that successful outcome of treatment will persist; however, we are planning to perform an examination of the patient in 3 years.

## Discussion and conclusions

The described type of kidney and ureteral triplication was identified as II type by Smith [1]. Occurrence of this type is 21% [6]. Existence of ectopic ureterocele negatively affects the upper moiety of triplicated kidney. Upper moiety function may be lost due to affected drainage and VUR. That may cause persistence of UTI and stones formation [7]. In this case there were significant pelvi-ureteric dilation and high grade VUR with UTI symptoms.

Contemporary trends in treatment of children with ureterocele mean conservative or minimally invasive surgical tactics with high possibility of getting good results. When we chose tactic of treatment we based on described criteria of patients selection for conservative treatment. These are lost function of upper moiety, absence of lower pole moieties obstruction, low grade VUR in the lower moiety ureter and absence of bladder neck obstruction [8]. There was no one mentioned criteria in this case. DMSA confirmed upper moiety function which was 23%. Conservative treatment was not indicated in this case and we could expect positive results of the use of minimal invasive surgical tactic. The patient had no associated congenital abnormalities which could discourage endoscopic surgery.

The second surgical procedure was necessary and successful. Decompression of ureterocele let to eliminate upper moiety dilation, VUR, urinary incontinence and UTI simultaneously. The first attempt to dissect ureterocele was unsuccessful probably because of minimal surgical impact on it.

This clinical case is an extremely rare one. In patients with large ureteral orifice in bladder neck and ureterocele the only applicable treatment is dissection of ureterocele. There is no need in orifice dilation like balloon plastic or bougieurage [9]. In case of ectopic ureteral orifice in bladder neck without ureterocele its dilation is acceptable [10]. Dissection of the front wall of ureterocele from the inside of it through the ectopic orifice allowed to create a new orifice of optimal size and position without risks of damage of deep bladder wall layers.

This extremely rare case of kidney triplication with ectopic ureterocele demonstrated complete improvement of the condition after endoscopic decompression of ureterocele.


**Additional file 1.** Video 1080 of this clinical case.


## Data Availability

All data generated or analyzed during this study are included in this published article and its supplementary information files and available from the corresponding author on reasonable request.

## Abbreviations

UTI: Urinary tract infection
IVP: Intravenous pyelogram
CT: Computer tomography
VUR: Vesicoureteral reflux
